# Surgery of the Knee

**Published:** 1919-08-02

**Authors:** 


### SURGERY OF THE KNEE.

### Notes of a Recent Development.

In recent years there has been an increasing tendency towards direct surgical treatment of looseness of the internal semilunar cartilage in the knee-joint, and personal experience of some fifteen cases inclines us to the opinion that such treatment is the best.

The operation as now performed is a perfect example of modern surgical technique, the procedure being bloodless, speedy, and with a very rapid prognosis of recovery. Assuming the patient to be fully anæsthetised, the affected limb is rendered avascular by means of a tourniquet applied about the upper part of the thigh. The limb now being fully flexed at the knee and partly everted, the mesial infrapatellar fossa is incised for a distance of three inches, the capsule being exposed and cut for a like distance, so that the semilunar cartilage is exposed and removed in its entirety.

The capsule and skin are now sewn up in one layer with salmon gut sutures, and a roll of broad bandage applied over a sterile dressing; this is still further reinforced by a roll of cotton wool, and then a further bandage is very firmly wound over all. The tourniquet is now removed, the tightness of the wool and its surrounding bandage being quite sufficient to prevent any oozing into the knee-joint from the cut surfaces.

The secret in the after-treatment rests in two essentials—early mobility and the confidence of the patient; one is no use without the other, for having previous experience of the agony of knee-joint pain, the patient will instinctively imagine that any early movement will cause pain. Thus on the day following operation, the patient is assured that only a little movement of the joint will be attempted, and that this will be painless. The whole limb is then raised from the hip-joint, the right hand being placed beneath the heel and the other under the popliteal space, a mobility of a few degrees is now secured by allowing the heel to drop slightly by its own weight, the whole manœuvre being carried out with great care, the slightest jerk or jar being avoided, as such would give a sharp twinge of pain. The patient is delighted with the painlessness of this slight movement, which is quite sufficient to prevent the possible formation of any adhesion between the healing capsule and the subjacent bones. On the second day the same movement is repeated, and on the third the patient is allowed to stand up with a "stiff knee," and then, whilst supported by the backs of two chairs, to bend the knee gradually: this secures two valuable results—the increasing mobility of the knee-joint and the avoidance of muscular atony, which would militate against the future stability of the joint by causing an undue laxity of the capsule and its surrounding musculature.

By the seventh day after the operation full mobility of the knee-joint is usually attained, and the stitches may be removed.

The knee-joint is now invested with an elastic bandage, which should be worn for the next month.

At the end of ten to fourteen days the patient is discharged from the surgeon's care, but is cautioned against any form of exercise which would entail any sudden violence to the knee-joint. Bicycle riding is an excellent after-treatment to develop and strengthen the muscles of the legs, and to still further secure the patient's confidence in the painless mobility of the joint.

Such after-treatment will appear very drastic to many practitioners, but the splendid results attained quite overshadow the results attained by the older and more conservative schools of surgery.

